# Incidence of ischemic stroke in Takotsubo cardiomyopathy patients: a systematic review and meta-analysis

**DOI:** 10.1186/s43044-026-00736-5

**Published:** 2026-04-12

**Authors:** Aleyda Zahratunany Insanitaqwa, Maya Qurota A’yun, Faizah Nadlirah, Aulia Putri Fadriyana

**Affiliations:** 1 University of Brawijaya, Malang, Indonesia; 2 Airlangga University, Surabaya, Indonesia

**Keywords:** Takotsubo cardiomyopathy, Stress-induced cardiomyopathy, Ischemic stroke, Cardioembolic stroke, Thrombotic stroke

## Abstract

**Background:**

Takotsubo cardiomyopathy (TC) is a cardiomyopathy characterized by temporary ventricular dysfunction, often resembling myocardial infarction. The association between ischemic stroke and TC remains unclear, with unknown true incidence. The objective of this study is to assess the incidence of ischemic stroke in TC patients and its risk factors.

**Methods:**

We carried out a comprehensive search of PUBMED, Google Scholar, PROQUEST, and ScienceDirect databases from their inception to February 2026. Studies reporting the incidence of ischemic stroke in TC patients were included. The quality of studies was evaluated utilizing the Joanna Briggs Institute Critical Appraisal Tools. Statistical analyses were performed using Review Manager and RStudio.

**Results:**

This study included six studies with 35,573 participants (841 had ischemic stroke and 34,732 did not). The pooled incidence of ischemic stroke was 4% (95% CI 0.01–0.06; I^2^ = 91%). The incidence of all-cause mortality was 36% (95% I 0.02–0.71, I^2^ = 81%) in TC patients with ischemic stroke, significantly higher than the 3.12% (95%CI:0.01–0.05, I^2^ = 37%) observed in TC patients without stroke. The mortality risk was higher in TC patients with ischemic stroke (OR7.57; 95% CI 2.70–21.22). Age (MD: 3.04; 95% CI 0.74–5.34), sex (OR0.64; 95% CI 0.53–0.77), diabetes (OR1.73; 95% CI 1.00–3.00), and atrial fibrillation (OR1.87; 95% CI 1.28–2.72) were significantly related to the incidence of ischemic stroke. However, smoking and hypertension showed no significant association with ischemic stroke.

**Conclusions:**

Ischemic stroke in TC patients correlates with an increased mortality rate and is associated with age, sex, and comorbidities, including diabetes and atrial fibrillation. Additional research is necessary to evaluate the possible advantages of anticoagulant therapy in TC patients.

**Supplementary Information:**

The online version contains supplementary material available at 10.1186/s43044-026-00736-5.

## Background

Takotsubo cardiomyopathy (TC) is a reversible cardiomyopathy that presents with clinical features resembling acute myocardial infarct, but without obstructive coronary artery disease or plaque rupture [1]. Approximately 2% of patients experiencing typical chest pain are diagnosed with TC, and it is found in about 2.5% of acute coronary syndrome [2, 3]. The condition is also known by several other names, including “apical ballooning syndrome”, “broken heart syndrome”, “stress-induced cardiomyopathy”, and “takotsubo syndrome” [4]. TC is characterized by a transient abnormality in the movement of the ventricular wall, particularly in the mid-distal and apical regions, accompanied by apical ballooning and a reduced ejection fraction. Recovery is usually spontaneous, occurring over the course of days or weeks [5]. The complication rate in TC patients varies from 22% to 50% [6]. Recent studies have highlighted an association between TC and thromboembolic complications, including stroke [7].

The incidence of stroke complications in TC ranges from 1% to 1.7% [4, 8]. There is ongoing debate among experts regarding whether ischemic stroke precipitates TC or whether TC leads to ischemic stroke, as well as how management strategies should address the prevention of both cardiac and cerebrovascular complications. Cases of TC occurring both prior to and following stroke have been reported. However, when these two conditions present concurrently, they may be attributed to a common stressor rather than one causing the other. Several studies have proposed a potential association between ischemic stroke and TC, although the underlying pathophysiology remains insufficiently understood [4, 8]. The proposed mechanism is primarily mediated by an acute catecholamine surge that induces myocardial stunning, transient regional wall-motion abnormalities, and endothelial dysfunction. These changes may promote intracavitary stasis and left ventricular thrombus formation, creating a substrate for cardioembolism and ischemic stroke. Catecholamine-related platelet activation and arrhythmias, including atrial fibrillation, may further increase thromboembolic risk. Conversely, acute neurological injury such as stroke can precipitate TC via excessive sympathetic activation, underscoring a bidirectional brain–heart interaction [7, 9]. Thromboembolic events have been reported in up to 15% of patients with TC [3].

Studies regarding the incidence of ischemic stroke in TC patients dominantly depend on case reports and observational studies that feature limited sample sizes. Up to this point, no systematic review or meta-analysis has been performed to evaluate the global incidence. This study is designed to assess the incidence of ischemic stroke in TC patients and to identify the determinants associated with it.

## Methods

### Data sources

We performed a systematic search across electronic databases including PubMed, Science Direct, Google Scholar, and PROQUEST from their inception to February 2026. The study was designed and carried out in accordance with the Preferred Reporting Item for Systematic Review and Meta-Analyses (PRISMA) guidelines and Cochrane Handbook for Systematic Reviews of Interventions. The keywords used in the search were “takotsubo cardiomyopathy” OR “takotsubo syndrome” OR “stress-related cardiomyopathy” OR “apical ballooning syndrome” OR “broken heart syndrome” AND “ischemic stroke” OR “thrombotic stroke” OR “cardioembolic stroke” OR “cerebrovascular accident”. The detailed search strategies for each database, including search terms and Boolean operators, are provided in Supplementary Table S1. References to all relevant original articles were manually reviewed to ascertain additional eligible studies. This study has been registered in PROSPERO (ID CRD42024628595).

### Eligibility criteria

Inclusion criteria were delineated as follows: (1) observational studies documenting the incidence of ischemic stroke in patients with TC; (2) studies involving adults aged 18 or more who were diagnosed with TC. We excluded studies if they were letters, review articles, case reports, or meta-analyses; not written in English; or duplicates. Ischemic stroke was confirmed using head computed tomography (CT)-scan or magnetic resonance imaging (MRI). Takotsubo cardiomyopathy was defined based on criteria established by the Mayo Clinic, which include four criteria that must be met: transient hypokinesis, akinesis or dyskinesis of the mid segments of the left ventricle, with or without involvement of the apex; absence of obstructive coronary disease or angiographic indicators of acute plaque rupture; presence of new electrographic abnormalities or modest increase in cardiac troponin; and absence of pheochromocytoma and myocarditis [2].

### Quality assessment and data extraction

Data extraction was performed independently based on pre-specified inclusion and exclusion criteria. The methodological quality of observational studies was evaluated using the Joanna Briggs Institute Critical Appraisal Tool [10]. Any uncertainties or missing data were addressed through discussion among the authors. Primary outcome measured was the incidence of ischemic stroke. Secondary outcomes measured were the mortality rate of ischemic stroke in TC patients and the association of comorbidities and ischemic stroke.

### Statistical analysis and data synthesis

All statistical analysis were performed using Review Manager 5.4 and RStudio. RevMan was used for primary meta-analysis and forest plot generation, while RStudio was utilized for additional analyses and to enhance data visualization and flexibility. A random-effects model was applied to all analyses. The mean differences (MDs) along with 95% confidence intervals (CIs) were computed for continuous data. The odds ratios (ORs) and 95% CIs were determined for dichotomous data. For single-arm outcomes, pooled proportions with corresponding 95% CIs were calculated. Heterogeneity was assessed using the I² statistic and Cochrane Q test. I² values of < 25%, 25–50%, and > 50% were considered to indicate low, moderate, and high heterogeneity, respectively. A p-value < 0.10 for the Cochrane Q test was considered statistically significant heterogeneity. Funnel plots were utilized to evaluate potential publication bias.

## Results

**Fig. 1:**
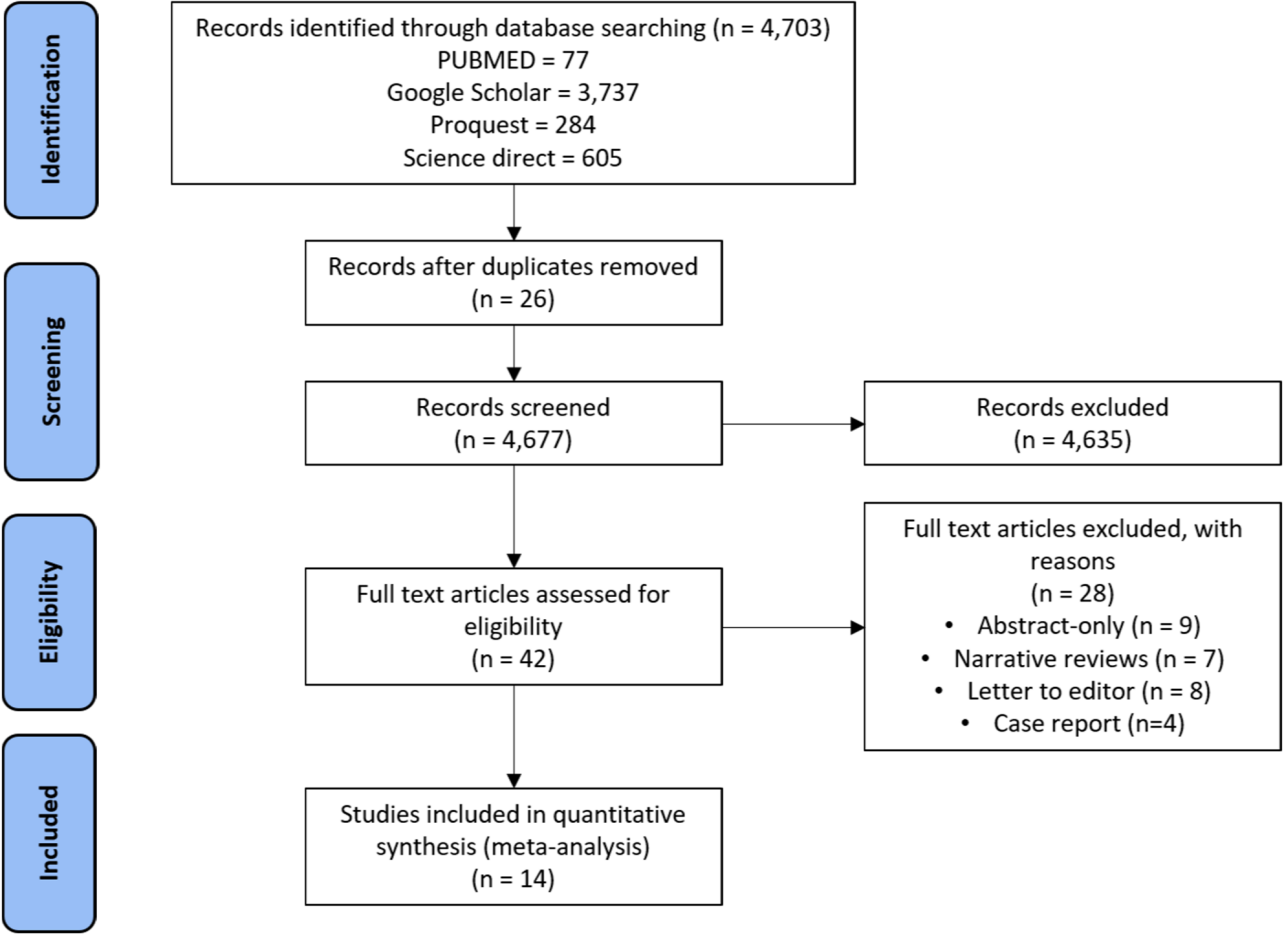
PRISMA flowchart depicting the number of studies identified, screened, and included in the analysis

The process of selecting data is illustrated in Fig. 1. This study included six studies with a total of 35,573 participants—841 with ischemic stroke and 34,732 without. The baseline characteristics of the studies are detailed in Table 1. The study designs primarily consisted of retrospective cohort study.

**Table 1:** Summary of the included studies

Author, year	Country	Study design	Period	Sample size (*n*)	Ischemic stroke [*n*, (%)]	Age (mean, SD)	Female [*n*, (%)]	Smoking status [*n*, (%)]	Comorbidities [*n*, (%)]	Length of stay (days)	Diagnosis	All-cause mortality [*n*]
Valbusa et al., 2008 [11]	Italia	Prospective cohort study	2001–2006	22	2 (9.1)	76.0 ± 7	22 (100.0)	0 (0.0)	- Dyslipidemia: 9 (40.9) - Hypertension: 18 (81.8) - DM: 4 (18.2) - Obesity: 7 (31.8)	N/A	- TC: ECG, echocardiography and coronary angiography - Stroke: unknown	2
Abanador-Kamper et al., 2018 [8]	Germany	Retrospective cohort study	2005–2017	71	3 (4.2)	68.8 ± 17.5	67 (94.4)	9 (12.7)	- Arterial hypertension: 49 (69.0) - DM: 7 (9.1) - Hyperlipidemia: 20 (28.2) - Obesity: 24 (33.8)	N/A	- TC: contrast-enhanced cardiac MRI - Stroke: brain MRI	− 1 after 30 days − 4 after 12 months
Dias et al., 2016 [3]	United States	Retrospective cohort study	2003–2014	206	15 (7.3)	67.8	179 (86.9)	83 (40.3)	- Hypertension: 141 (68.4) - Hyperlipidemia: 92 (44.7) - DM: 41 (19.9) - Atrial Fibrillation: 17 (8.3)	9	- TC: 12-lead ECG, echocardiography, and cardiac catheterization - Stroke: brain CT-scan	15 - No stroke : 12 - Stroke : 3
Mitsuma et al., 2008 [5]	Japan	Cross-sectional study	1999–2008	21	2 (9.5)	72 ± 5	18 (85.7)	N/A	N/A	N/A	- TC: echocardiography and 12-lead ECG - Stroke : unknown	0
Morris et al., 2020 [9]	United States	Retrospective cohort study	2005–2015	5,283	68 (1.3)	67.1 ± 13	4,808 (91.0)	- Stroke : 13 (19.1) - No stroke : 865 (16.6)	Stroke vs. no stroke - Hypertension: 49 (72.1) vs. 3,433 (65.8) - DM: 27 (39.7) vs. 1,112 (21.3) - Atrial fibrillation: 18 (26.5) vs. 621 (11.9)	N/A	- TC and stroke: validated ICD-9-CM diagnosis code on medical records	N/A
Abe et al., 2021 [7]	United States	Cross-sectional study	2008–2017	29,970	751 (2.5)	- No stroke: 67 ± 13 - Stroke: 69 ± 13	- No stroke: 24,007 (88.2) - Stroke: 620 (82.3)	- No stroke: 4,763 (17.5) - Stroke : 142 (18.9)	Stroke vs. no stroke - Hyperlipidemia: 364 (48.4) vs. 11,976 (44.0) - Obesity: 64 (8.5) vs. 2,857 (10.5) - Hypertension: 87 (11.6) vs. 3,674 (13.5) - DM: 35 (4.7) vs. 1,034 (3.8)	- No stroke : 3 - Stroke : 7	- TC: coronary angiography - TC and stroke: ICD-9-CM and ICD-10-CM	Stroke : 96 No Stroke : 730

*CT-scan* computerized tomography scan, *DM* diabetes mellitus, *ECG* electrocardiogram, *ICD-9-CM* International Classification of Diseases Ninth Revision Clinical Modification, *ICD-10-CM* International Classification of Diseases Tenth Revision Clinical Modification, *MRI* magnetic resonance imaging, *N/A* not applicable, *SD* standard deviation, *TC* Takotsubo cardiomyopathy,

### Ischemic stroke

**Fig. 2:**
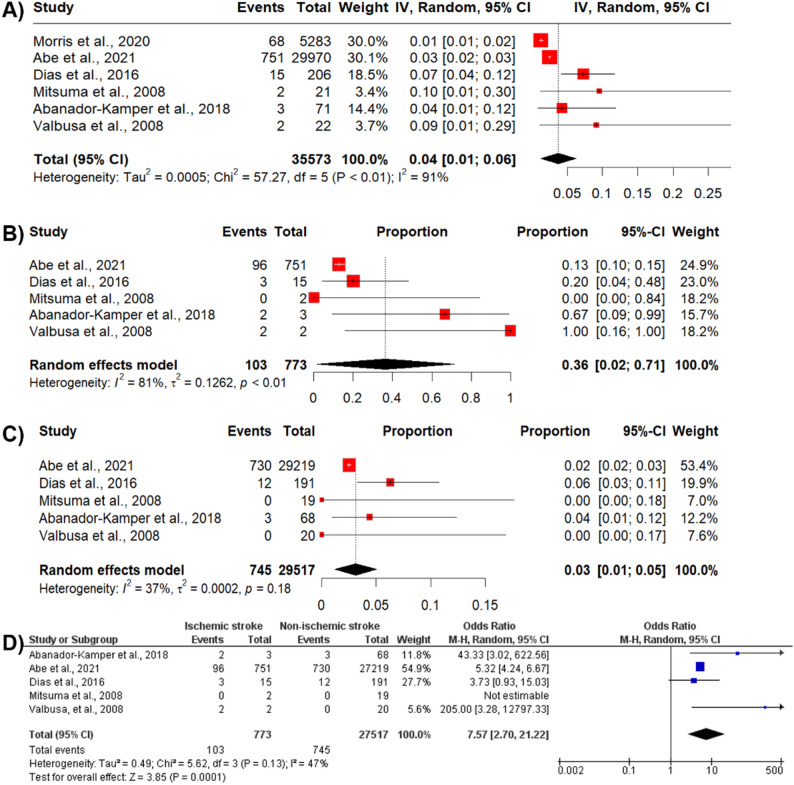
Incidence and mortality of TC patients in relation to ischemic stroke. **A** Incidence of ischemic stroke among TC patients. **B** Mortality rate among TC patients with ischemic stroke. **C** Mortality rate among TC patients without ischemic stroke. **D** Comparison of mortality rates between TC patients with and without ischemic stroke

The total number of patients with ischemic stroke across all studies was 841. The pooled incidence of ischemic stroke among patients with TC was 4% (95% Confidence Interval [CI]: 1.00–6.00). However, high heterogeneity was noted among the included studies (I^2^ = 91%, *p* < 0.01) (Fig. 2A).

### Mortality rate

The number of deaths among TC patients without ischemic stroke was 29,517, with an incidence rate of 3% (95% CI 0.01–0.05, I^2^ = 37%, *p* = 0.18). In contrast, the total number of deaths among TC patients with ischemic stroke was 773, with an incidence rate of 36% (CI 2.00–7.10, I^2^ = 81%, *p* < 0.01)), which was higher than that of patients without ischemic stroke. A statistically significant difference was observed in the odds of mortality between the ischemic stroke group and the non-ischemic stroke group (Odd ratio [OR] 7.57; 95% CI 2.70–21.22, *p* = 0.0001). Publication bias assessment of mortality among TC patients with ischemic stroke using Doi plot showed no asymmetry (LFK index = − 0.45), while among TC patients without ischemic stroke demonstrated major asymmetry (LFK index = − 2.24) (see Supplementary Fig. S2 and S3).

### Comorbidities

**Fig. 3:**
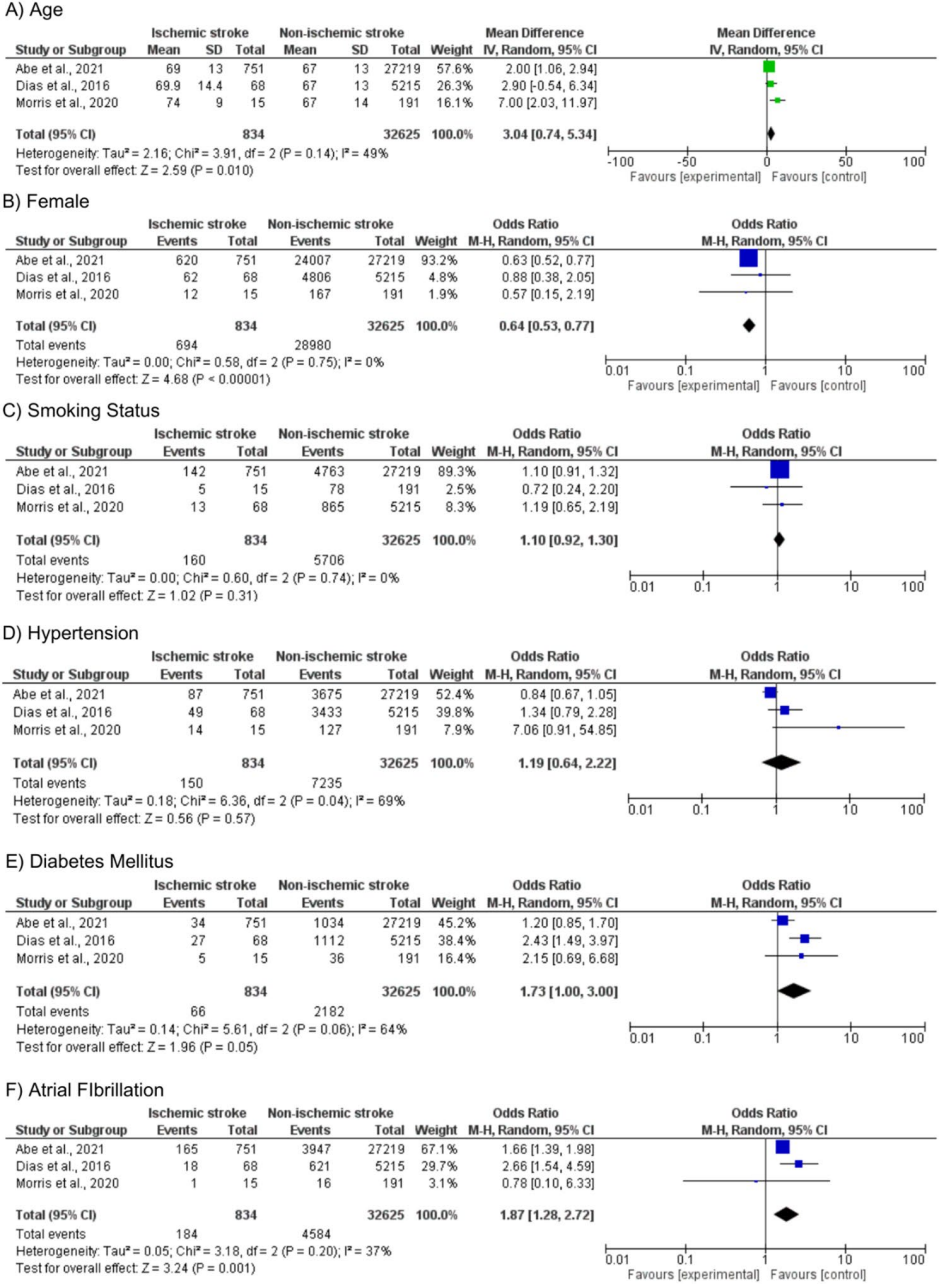
Forest plots comparing comorbidities between TC patients with and without ischemic stroke. Panels show age (**A**), female sex (**B**), smoking status (**C**), hypertension (**D**), diabetes mellitus (**E**), and atrial fibrillation (**F**)

Age (MD: 3.04; 95% CI 0.74–5.34; *p* = 0.01), sex (OR: 0.64; 95% CI 0.53–0.77; *p* < 0.01), diabetes mellitus (OR 1.73; 95% CI 1.00–3.00; *p* = 0.05), and atrial fibrillation (OR 1.87; 95% CI1.28–2.72; *p* = 0.001) were significantly related to the incidence of ischemic stroke. In contrast, smoking status (OR: 1.10; 95% CI 0.92–1.30; *p* = 0.31) and hypertension (OR: 1.19; 95% CI 0.64–2.22; *p* = 0.57) did not show a significant association with the incidence of ischemic stroke (Fig. 3). Doi plot analysis demonstrated minor asymmetry for age, female sex, and diabetes mellitus (LFK index = 1.73 for each), suggesting a possible small-study effect. In contrast, smoking status, hypertension, and atrial fibrillation showed no evidence of asymmetry (LFK index = 0.58 for each). These results should be interpreted with caution given the limited number of included studies.

## Discussion

In this meta-analysis, the incidence rate of ischemic stroke among patients with TC was found to be 4%. However, previous observational studies have reported heterogenous incidence rates of ischemic stroke among TC patients. One study reported a 1.7% incidence, while a cohort study found that 1.3% of TC patients experienced stroke within the first two years [9]. Based on data of International Takotsubo Registry (InterTAK), the incidence of ischemic stroke was 1.3% in the first 30-day and 1.7% per patient-year in the first 10 year follow-up [12]. These discrepancies likely reflect differences in study populations (e.g., age, sex, and comorbidity burden), diagnostic criteria, follow-up duration, and methodological approaches, as well as temporal changes in clinical practice. By synthesizing data from multiple studies, our meta-analysis provides a more robust and comprehensive estimate of the overall risk of ischemic stroke in patients with TC.

Several studies have suggested a bidirectional association between TC and ischemic stroke. Acute ischemic stroke may trigger a catecholamine surge via activation of the hypothalamic-pituitary-adrenal axis, leading to myocardial injury, transient ventricular dysfunction, and endothelial impairment [4]. Catecholamine surge also inhibit Gαi activity through “stimulus trafficking”, which leads to decreased inotropy, chronotropy, and lusitropy, with apical hypokinesia [2]. These changes can promote intracavitary stasis and thrombus formation, increasing the risk of cardioembolic stroke. Conversely, TS itself may predispose to ischemic stroke through hypercoagulability, platelet activation, and reduced cardiac output [4]. Damage to specific brain regions, particularly the insular cortex and autonomic centers, may disrupt sympathetic–parasympathetic balance, contributing to cardiac dysfunction and arrhythmias [3, 4, 13–15]. In addition, abnormalities in ventricular wall motion in TS can lead to left ventricular thrombus formation, which has been reported in up to 5–8% of patients, representing an important mechanism linking TC to ischemic stroke [9, 16].

More recent evidence suggests that both TS and ischemic stroke may arise from shared underlying mechanisms, particularly microvascular dysfunction. Impaired microcirculation, endothelial dysfunction, and inflammatory activation may affect both cardiac and cerebral perfusion. Catecholamine excess may further exacerbate oxidative stress and vascular injury, providing a plausible link between these two conditions [17].

Female sex is considered a major risk factor for TC, particularly in postmenopausal women [18]. Approximately 90% of TC cases occur in postmenopausal women. Estrogen provides cardioprotection by decreasing the sensitivity of beta-1 adrenergic receptors, preventing cardiac myocyte apoptosis, lowering intracellular calcium levels, boosting atrial natriuretic peptide, and inhibiting NF-kB activation. Additionally, estrogen plays a role in regulating vasomotor tone by enhancing endothelial nitric oxide synthase, which helps reduce vasoconstriction and sympathetic stress response in older women [4]. In 2019, stroke was the third leading cause of death among women and the fifth leading cause among men. For individuals aged 55 and older, women encounter a higher lifetime risk of stroke (20–21%) in comparison to men (14–17%) [19]. However, our findings suggest that women are less likely to experience ischemic stroke and further research is needed to confirm these findings.

While TC mainly affects postmenopausal women, it can also occur in younger individuals. The link between age and short-term outcomes in TC is still debated. Some studies suggest that older patients have higher short-term mortality rates, including in-hospital deaths, due to reduced cardiac vagal tone and increased sympathetic activation in older individuals. On the other hand, other research suggested that younger patients may face more inpatient complications, including the need for invasive ventilation, cardiopulmonary resuscitation, catecholamine, cardiogenic shock, and ventricular arrhythmia. There are also studies that find no clear association between age and short-term mortality. However, the majority of research showed that older TC patients tend to have worse long-term outcomes compared to younger patients [18, 20, 21]. Additionally, stroke risk varies by age and sex. Stroke incidence is higher in women under 30, but during midlife, men have higher rates. From the age of 80 onward, stroke rates are either similar or higher in women [19]. This study found that those who develop ischemic stroke are, on average, 3 years older, with statistically significant differences.

Approximately 22% of TC patients are smokers [2]. A multicenter study revealed that smoking is linked to non-apical TC. While smokers experienced similar rates of in-hospital complications as non-smokers, they had longer hospital stays. Additionally, they were at a higher risk of developing a stroke and requiring orotracheal intubation. However, no significant differences in long-term mortality rate were found between the two groups. Both smoking and TC are known to cause oxidative stress in cardiomyocytes, which can lead to myocardial dysfunction [22]. However, this study did not find a strong association between smoking and ischemic stroke.

Approximately 82% of TC patients have hypertension. Hypertension causes endothelial dysfunction and vascular remodeling, primarily through reduced nitric oxide. This condition leads to low-grade inflammation, vascular fibrosis, microvascular rarefaction, and an enhanced myocardial vessel response to catecholamines [11]. Abnormal vasomotor regulation in hypertension results in vasoconstriction and diminished vasodilatory responses. Hypertensive patients also exhibit an increased catecholamine response to stressors. In a study by Dias et al., hypertension was recognized as an independent predictor of stroke in TC patients [3]. The risk of stroke related to hypertension could be as high as 50% in specific racial and ethnic groups [23]. Nonetheless, this meta-analysis did not establish a significant correlation between hypertension and ischemic stroke.

While diabetes mellitus (DM) is a recognized risk factor for cardiovascular disease, its association with TC remains uncertain [18]. Diabetic TC patients encounter a 16–25% increased risk of experiencing thrombotic stroke [7]. Nevertheless, numerous studies have indicated that DM does not significantly influence the prognosis of TC. One possible explanation is that TC patients with DM may have lower levels of catecholamine release due to diabetic autonomic neuropathy, which could provide some protection against complications such as stroke [18]. This study also found a significant link between diabetes and ischemic stroke in TC patients.

Atrial fibrillation (AF) is present in 6.4% to 25.06% of patients with TC. A meta-analysis demonstrated that atrial dysfunction doubles all-cause mortality rate in patients with TC compared to those without TC. The development of AF in TC may be influenced by left atrial dysfunction and excessive catecholamine release [24]. AF in TC patients is linked to cardioembolic stroke, as reduced ventricular function and atrial enlargement can cause blood stasis and thromboembolism [7]. Despite this, only 1% of TC patients with AF die from stroke [2]. This study also indicated that AF constitutes a significant risk factor for ischemic stroke in this population.

The 6-month mortality rate for patients with TC is reported to be 7.5% [25]. However, a cohort study comparing TC patients to those with myocardial infarction found that, over a 5-year period, TC patients had a similar mortality rate but a lower risk of cardiovascular rehospitalization [26]. Additionally, in this study, we found that TC patients experience a significantly higher mortality rate (36%) compared to those without ischemic stroke (3%). Despite this stark contrast, there is substantial heterogeneity in the reported mortality rates, suggesting variability in the outcomes based on patients characteristics, comorbidities, and/or management. Furthermore, in-hospital mortality rates for TC patients who experience ischemic stroke have remained consistent over the past decade, potentially attributable to the absence of established guidelines for the prevention and management of ischemic stroke among TC patients. This highlights the need for further research and better clinical protocols for managing TC with ischemic stroke [7].

Based on our findings, risk stratification for ischemic stroke in patients with TC is essential. One study demonstrated that the CHA₂DS₂-VASc score is a significant predictor of adverse in-hospital outcomes, including mortality, ischemic stroke, intracardiac thrombus formation, cardiogenic shock, and the need for renal replacement therapy. Higher CHA₂DS₂-VASc scores were consistently associated with an increased risk of these clinical events. These results support the utility of the CHA₂DS₂-VASc score as a practical tool for early risk assessment in patients with TC [27].

Our study suggests that appropriate anticoagulation treatment should be provided to all TC patients to avert embolic events, such as ischemic stroke, until any wall motion abnormalities improve. At present, there are no definitive guidelines regarding anticoagulation therapy in TC patients, given that the condition is reversible and generally self-limiting [8, 15]. One study proposed anticoagulant therapy for individuals at higher risk of stroke, particularly those exhibiting an apical ballooning pattern and elevated troponin-I levels exceeding 10 ng/mL. For these individuals, the presence of LV thrombus should prompt anticoagulant therapy for a minimum duration of 3 months. Nonetheless, for patients exhibiting midventricular or basal ballooning, or apical ballooning with troponin-I levels less than 10ng/ml, the use of oral anticoagulant should not be undertaken [28]. Follow-up using echocardiography and clinical features are needed, to decide the duration of anticoagulant therapy. Duration of anticoagulant can be decided based on patients’ benefit-risk ratio [5, 11, 29]. Low molecular weight heparin, unfractionated heparin, direct oral anticoagulants, or vitamin K antagonist are the preferred anticoagulant [2, 29]. Heparin should be administered in the presence of thrombus, followed by warfarin until at follow-up the thrombus has resolved, with some additional aspirin [29, 30]. Aspirin and clopidogrel, which have protective cardiovascular effects, could be the approaches targeting catecholamines and inflammatory biomarkers [3]. Further investigations, specifically randomized trials, are essential to assess the effectiveness of anticoagulation therapy in TC patients.

There are several limitations in this study. All studies included were observational; therefore, the effect of residual confounders could not be entirely disregarded. Moreover, the observational characteristics of the studies contributed some heterogeneity, which diminished the statistical power to predict the association between ischemic stroke and TC. The relatively small number of included studies limited our ability to perform additional analyses, such as sensitivity analysis. Additionally, research published in languages aside from English were not included. Finally, the limited number of relevant articles available may have impacted the outcomes. Additional studies with larger sample sizes are necessary to produce more reliable conclusions.

## Conclusion

In summary, TC patients may experience ischemic stroke and have an increased mortality rate compared to those without stroke. This is influenced by factors such as age, sex, and comorbidities like diabetes mellitus and atrial fibrillation. Our findings indicate possible benefits for oral anticoagulation in high-risk TC patients until improvement of left ventricular function.

## Supplementary Information

Below is the link to the electronic supplementary material.

Supplementary Material 1.

## Data Availability

No datasets were generated or analysed during the current study.

## Abbreviations

AF: Atrial fibrillation
CI: Confidence interval
CT-scan: Computed tomography scan
DM: Diabetes mellitus
ECG: Electrocardiogram
ICD-9-CM: International Classification of Diseases, Ninth Revision, Clinical Modification
ICD-10-CM: International Classification of Diseases, Tenth Revision, Clinical Modification
InterTAK: International Takotsubo Registry
LVEF: Left ventricular ejection fraction
LVT: Left ventricular thrombus
MD: Mean differences
MRI: Magnetic resonance imaging
N/A: Not applicable
OR: Odds ratio
PRISMA: Preferred Reporting Item for Systematic Review and Meta-Analyses
SD: Standard deviation
TC: Takotsubo cardiomyopathy
