# Supplementary material for: Incidence of ischemic stroke in Takotsubo cardiomyopathy patients: a systematic review and meta-analysis
Source: Egypt Heart J. 2026 Apr 12;78:23. doi: 10.1186/s43044-026-00736-5 (PMC13070880; doi:10.1186/s43044-026-00736-5)
Supplement: Supplementary file 1 — Supplementary Material 1. [file 43044_2026_736_MOESM1_ESM.docx]

SUPPLEMENTARY FILES

**INCIDENCE OF ISCHEMIC STROKE IN TAKOTSUBO CARDIOMYOPATHY PATIENTS: A SYSTEMATIC REVIEW AND META-ANALYSIS**

**Supplementary Table 1:** Search Strategy

| **Database** | **Search Strategy** |
| --- | --- |
| PubMed | ("Takotsubo Cardiomyopathy"[Mesh] OR "takotsubo cardiomyopathy"[Title/Abstract] OR "takotsubo syndrome"[Title/Abstract] OR "stress-induced cardiomyopathy"[Title/Abstract] OR "apical ballooning syndrome"[Title/Abstract] OR "broken heart syndrome"[Title/Abstract])  AND  ("Stroke"[Mesh] OR "ischemic stroke"[Title/Abstract] OR "thrombotic stroke"[Title/Abstract] OR "cardioembolic stroke"[Title/Abstract] OR "cerebrovascular accident"[Title/Abstract]) |
| ScienceDirect | ("takotsubo cardiomyopathy" OR "takotsubo syndrome" OR "stress-induced cardiomyopathy" OR "apical ballooning syndrome" OR "broken heart syndrome")  AND  ("ischemic stroke" OR "thrombotic stroke" OR "cardioembolic stroke" OR "cerebrovascular accident")  Filters applied: research articles, English language. |
| ProQuest | ("takotsubo cardiomyopathy" OR "takotsubo syndrome" OR "stress-induced cardiomyopathy" OR "apical ballooning syndrome" OR "broken heart syndrome")  AND  ("ischemic stroke" OR "thrombotic stroke" OR "cardioembolic stroke" OR "cerebrovascular accident")  Filters applied: peer-reviewed articles, English language. |
| Google Scholar | ("takotsubo cardiomyopathy" OR "takotsubo syndrome") AND ("ischemic stroke" OR "cerebrovascular accident") |

**Supplementary Table 2:** JBI Critical Appraisal Checklist for Studies Reporting Prevalence Data

| **Study** | **1. Was the sample frame appropriate to address the target population?** | **2. Were study participants sampled in an appropriate way?** | **3. Was the sample size adequate?** | **4. Were the study subjects and the setting described in detail?** | **5. Was the data analysis conducted with sufficient coverage of the identified sample?** | **6. Were valid methods used for the identification of the condition?** | **7. Was the condition measured in a standard, reliable way for all participants?** | **8. Was there appropriate statistical analysis?** | **9. Was the response rate adequate, and if not, was the low response rate managed appropriately?** | **Included study (Y/N)** |
| --- | --- | --- | --- | --- | --- | --- | --- | --- | --- | --- |
| Morris et al., 2020 | Yes | Yes | Yes | Yes | Yes | Yes | No | yes | N/A | Yes |
| Abe et al., 2021 | Yes | Yes | Yes | Yes | Yes | Yes | Yes | Yes | Yes | Yes |
| Dias et al., 2016 | Yes | Yes | No | Yes | Yes | Yes | Yes | Yes | Yes | Yes |
| Mitsuma et al., 2008 | Yes | Yes | No | Yes | Yes | Yes | Yes | No | N/A | Yes |
| Abanador-Kamper et al., 2018 | Yes | Yes | No | Yes | Yes | Yes | Yes | Yes | N/A | Yes |
| Valbusa et al., 2008 | Yes | Yes | No | Yes | Yes | Unclear | Yes | No | N/A | Yes |

N/A = Not applicable

**Supplementary Figure 2:** Funnel and DOI Plots

1. Incidence of ischemic stroke in TC patients


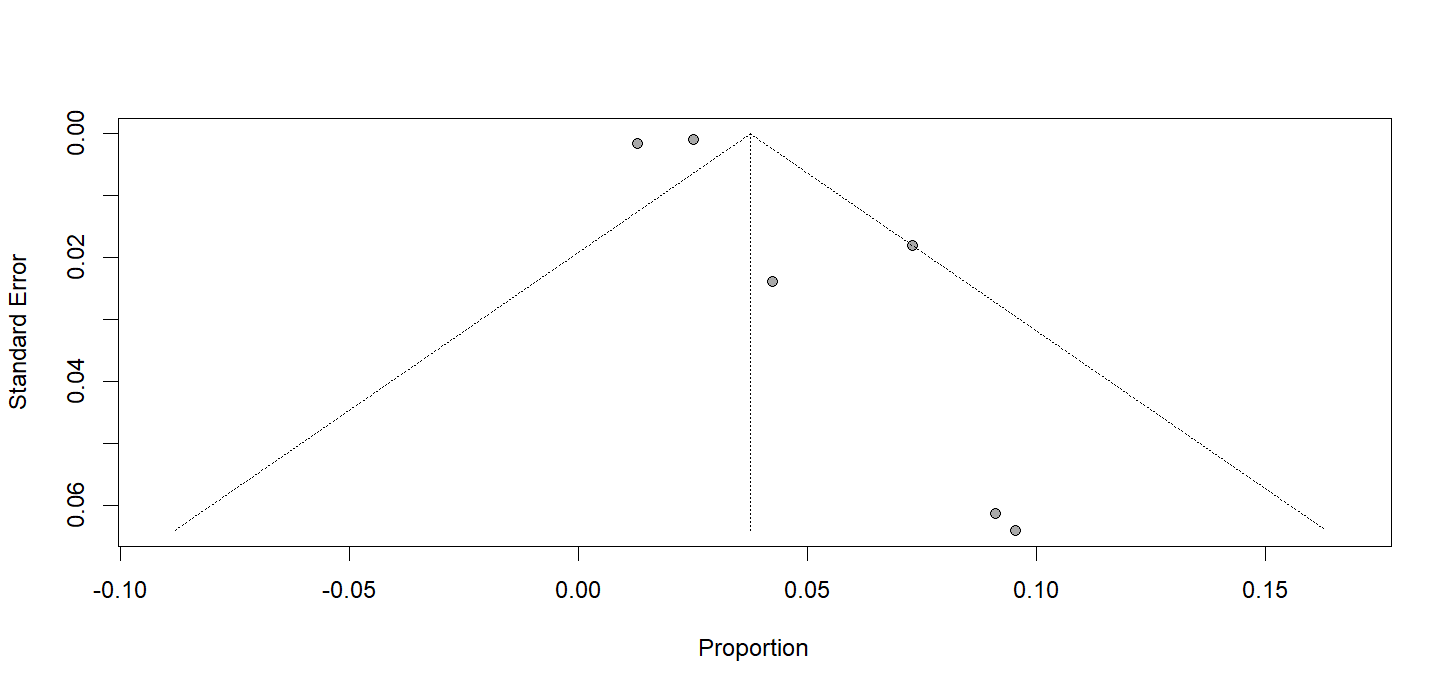


**Figure S1.** Funnel plot assessing publication bias for incidence of ischemic stroke among Takotsubo cardiomyopathy patients.

1. Mortality rate in TC patients with ischemic stroke


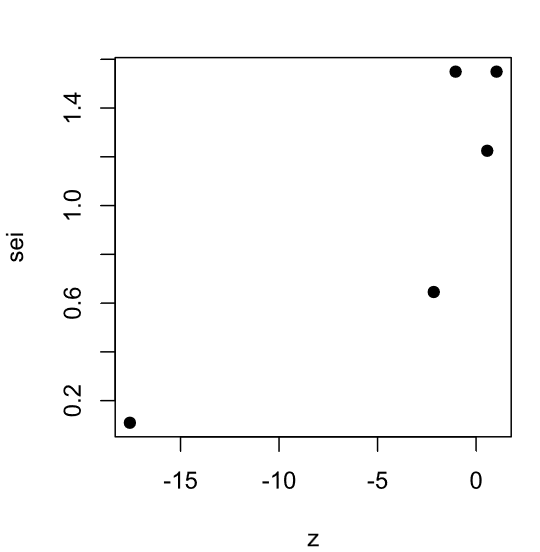


**Figure S2.** Doi plot assessing publication bias for mortality among Takotsubo cardiomyopathy patients with ischemic stroke. The plot demonstrates a symmetrical distribution of studies, indicating no evidence of asymmetry (LFK index = −0.45).

1. Mortality rate in TC patients without ischemic stroke


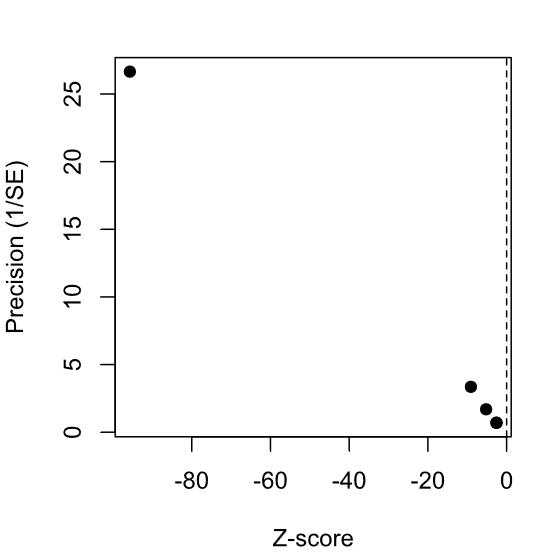


**Figure S3.** Doi plot assessing publication bias for mortality among Takotsubo cardiomyopathy patients with ischemic stroke. The plot demonstrates a major asymmetry distribution of studies, indicating potential publication bias or small-study effects (LFK index = -2.24).

1. Comparing age in ischemic stroke vs non-ischemic stroke


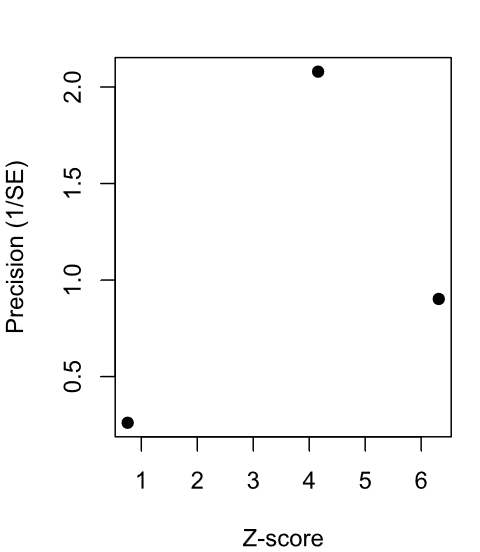


**Figure S4.** The Doi plot for age demonstrated minor asymmetry (LFK index = 1.73).

1. Comparing sex (female) in ischemic stroke vs non-ischemic stroke


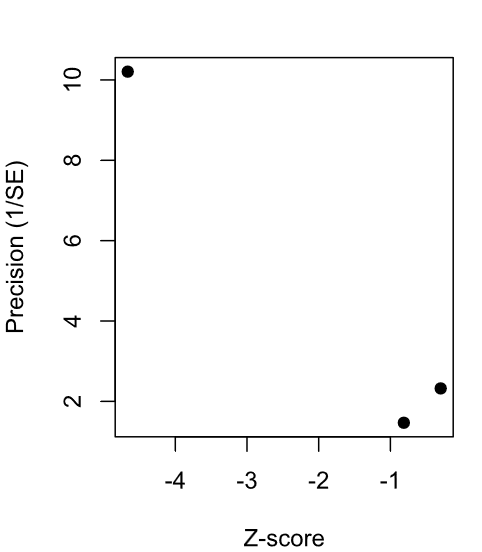


**Figure S5.** The Doi plot for female demonstrated minor asymmetry (LFK index = 1.73).

1. Comparing smoking status in ischemic stroke vs non-ischemic stroke


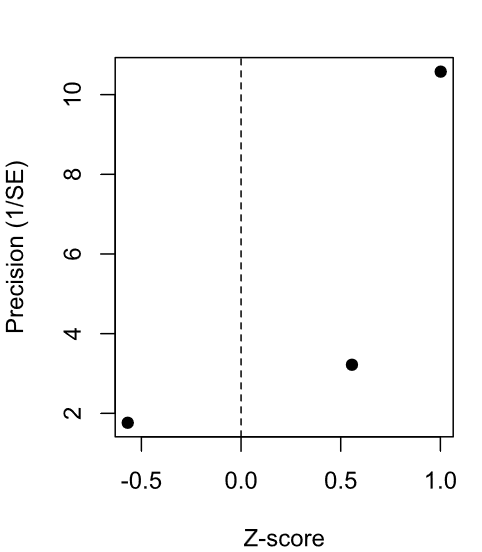


**Figure S6.** The Doi plot for smoking status demonstrated no asymmetry (LFK index = 0.58).

1. Comparing hypertension in ischemic stroke vs non-ischemic stroke


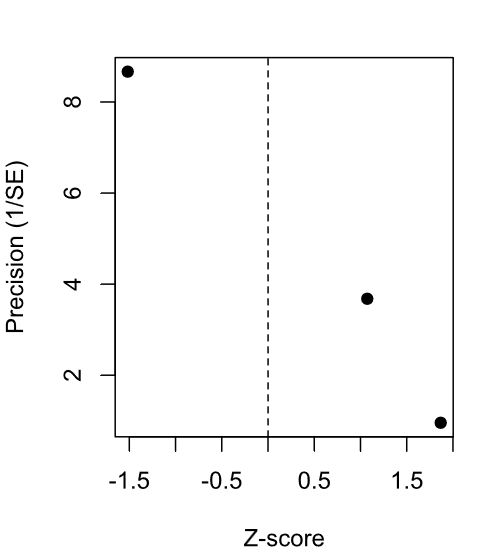


**Figure S7.** The Doi plot for hypertension demonstrated no asymmetry (LFK index = 0.58).

1. Comparing diabetes mellitus in ischemic stroke vs non-ischemic stroke


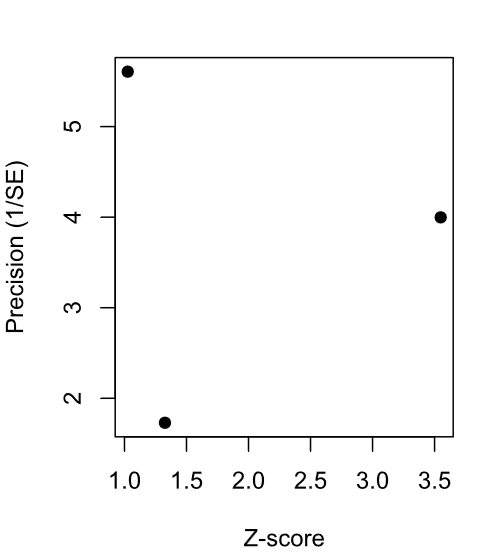


**Figure S8.** The Doi plot for diabetes mellitus demonstrated minor asymmetry (LFK index = 1.73).

1. Comparing atrial fibrillation in ischemic stroke vs non-ischemic stroke


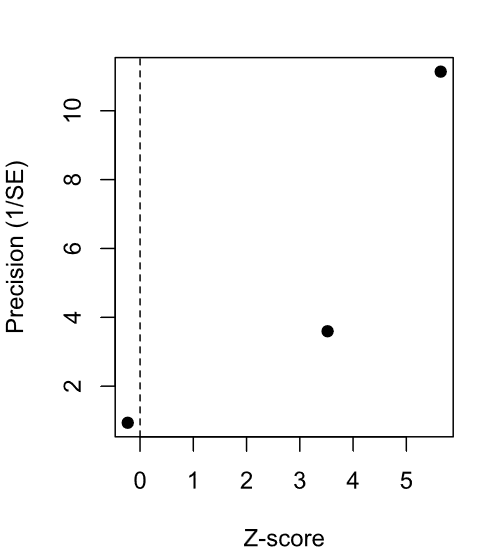


**Figure S9.** The Doi plot for atrial fibrillation demonstrated no asymmetry (LFK index = 0.58).
